# Laboratory Surveillance of *Acinetobacter* spp. Bloodstream Infections in a Tertiary University Hospital during a 9-Year Period

**DOI:** 10.3390/tropicalmed8110503

**Published:** 2023-11-19

**Authors:** Anastasia Spiliopoulou, Ioanna Giannopoulou, Stelios F. Assimakopoulos, Eleni Jelastopulu, Christina Bartzavali, Markos Marangos, Fotini Paliogianni, Fevronia Kolonitsiou

**Affiliations:** 1 Department of Microbiology, University Hospital of Patras, 265 04 Rio, Greecechris.bartzavali@gmail.com (C.B.); fpal@upatras.gr (F.P.); kolonits@upatras.gr (F.K.); 2 Department of Infectious Diseases, University Hospital of Patras, 265 04 Rio, Greece; sassim@upatras.gr (S.F.A.); mmarangos@yahoo.com (M.M.); 3 Department of Public Health, School of Medicine, University of Patras, 265 04 Patras, Greece; ejela@upatras.gr

**Keywords:** *Acinetobacter*, *A. baumannii*, bloodstream infections, ICU infections, tigecycline, colistin, carbapenems, multidrug-resistant, extensively drug-resistant, pandrug-resistant

## Abstract

Multidrug-resistant *Acinetobacter baumannii* infections have become a threat for public health worldwide. The aim of the present study was to follow-up resistance patterns of *Acinetobacter* spp. bloodstream isolates in a Tertiary University Hospital over the last nine years, from 2014 to 2022. Susceptibility patterns were followed for the following antimicrobial agents: amikacin, gentamicin, tobramycin, ciprofloxacin, levofloxacin, imipenem, meropenem, tigecycline, trimethoprim/sulfamethoxazole, and colistin. Minimal inhibitory concentration (MIC) values to ampicillin/sulbactam, cefepime, ceftazidime, minocycline, piperacillin/tazobactam were evaluated from 2020 to 2023. During the study period, 853 *Acinetobacter* spp. bloodstream infections (BSIs) were recorded, accounting for 5.36% of all BSIs. *A*. *baumannii* was isolated in 795 cases (93.2%), during the study period. Most BSIs were recorded in adult intensive care units (ICU) (46.2%) and medical wards (42%). Among *A. baumannii* isolates, 4.5% were multidrug-resistant, 84.7% were extensively drug-resistant, and 8.5% were pandrug-resistant. Resistance to carbapenems was over 95%. Resistance to tigecycline increased significantly during the last years of the study (2020–2022); *A. baumannii* isolates with MIC ≤ 2 μg/mL accounted for 28.5% of all isolates. Resistance to colistin exhibited an increasing pattern up to 42.2% in 2022. Increasing resistance rates and the evolution of pandrug-resistant isolates call for the urgent application of preventive and response actions.

## 1. Introduction

The *Acinetobacter* genus has undergone significant taxonomic modification over the last 30 years [1]. To date, 74 *Acinetobacter* species have been nominated [2]. *A. baumannii* is the species most commonly isolated from human infections [2] and represents one of the most problematic pathogens for healthcare institutions globally [1]. *Acinetobacter* spp. remain stable in a hospital environment, can easily colonize inpatients and staff, and cause outbreaks, particularly in intensive care units (ICUs) [3]. Apart from its inherent resistance to many antimicrobials, acquired resistance further complicates the treatment of serious infections in already vulnerable patient groups [1,3]. Furthermore, its ability for biofilm formation promotes its success as a nosocomial pathogen by adhering to catheters and ventilators causing central line-associated bloodstream infections and ventilator-associated pneumonia [4]. Fundamental virulence factors, such as surface adhesins, glycoconjugates, and secretion systems directly contribute to the pathogenesis of *A. baumannii* infections [4]. More specifically, the outer membrane protein A as the main virulent factor of outer membrane vesicles may cause mitochondrial fragmentation and death of host epithelial cells and macrophages [5]. Virulence traits including biofilm formation, pellicle formation, motility, and resistance are sophisticatedly controlled by regulatory systems, such as a two-component regulatory system and quorum sensing system, which allow the pathogen to survive in harsh environments and infect susceptible hosts [6,7].

Due to its large public-health implications, the World Health Organization (WHO) ranked carbapenem-resistant *A. baumannii* as a “critical-priority” pathogen for investment in research and development of effective drugs [8]. In 2019, the Centers for Disease Control and Prevention (CDC) published its second report on antibiotic resistance threats in the United States and categorized carbapenem-resistant *A. baumannii* as an urgent threat, thus prompting continual public-health monitoring and prevention activities [9]. Resistance to antibiotics gives this bacterium, along with *Enterococcus faecium*, *Staphylococcus aureus*, *Klebsiella pneumoniae*, *Pseudomonas aeruginosa*, and *Enterobacter* species, a place among the nosocomial ESKAPE pathogens [10].

*Acinetobacter* BSIs commonly have poor outcomes, especially in ICU patients [1]. In a large study of nosocomial BSIs in the United States (1995–2002), *A. baumannii* was the 10th most common etiologic agent, and the crude mortality overall from *A. baumannii* BSIs was 34.0% to 43.4% in the ICU [1]. Moreover, globally, ~45% of all isolates are multidrug-resistant (MDR), with rates as high as 70% in Latin America and the Middle East [4]. Carbapenem-resistance is widespread, with rates exceeding 90% in some Southern and Eastern European countries [11]. Due to the limited therapeutic options, colistin is a commonly used treatment in critically ill patients. However, colistin-resistant *A. baumannii* isolates have been recorded worldwide, with resistance rates reaching 12% in China, and 17% in Lebanon [11]. In a recent study conducted in Europe, the percentage of colistin-resistant *A. baumannii* isolates was 8% [12]. However, in Greece and Lithuania resistance rates up to 54.6 and 18.3%, respectively, have been recorded between 2020 and 2021 [12]. A previous study conducted in our settings, from 2006 to 2013, revealed increasing resistance rates of *A. baumannii* isolated from BSIs to meropenem (up to 83.3%), tigecycline (66.5%), and minocycline (40.3%). Interestingly, colistin-resistant isolates were not recorded [13]. All these data highlight the requirement for reinforced *Acinetobacter* surveillance as a part of infection prevention and control. The aim of the present study was to follow up antimicrobial resistance patterns of *Acinetobacter* spp. bloodstream isolates over the past nine years, from 2014 to 2022.

## 2. Materials and Methods

### 2.1. Study Design

In the present study, all *Acinetobacter* spp. bloodstream isolates from patients hospitalized in the University General Hospital of Patras in Southwestern Greece, from January 2014 to December 2022, were recorded. Patients’ records were retrieved from four main sectors: Medical Wards (MW) including Internal Medicine, Cardiology, Nephrology, Neurology, Haematology–Oncology and Hematopoietic Stem Cell Transplantation Unit; Surgical Wards (SW) including General Surgery Unit, Orthopedics, Obstetrics, Neurosurgery and Urology; adult Intensive Care Unit (ICU); Neonatal Intensive Care Unit (NICU) and Pediatric Intensive Care Unit (PICU). Subsequent isolation of *Acinetobacter* spp. from the same patient was not considered a new bloodstream infection (BSI) unless there was an interval ≥14 days [14].

### 2.2. Bacterial Identification and Antimicrobial Susceptibility Testing

Identification of *Acinetobacter* spp. was performed by VITEK^®^ 2 Gram-negative identification cards (bioMérieux, Marcy-l’Etoile, France). Susceptibility to the following antimicrobial agents was studied: amikacin, gentamicin, tobramycin, ciprofloxacin, levofloxacin, imipenem, meropenem, tigecycline, trimethoprim/sulfamethoxazole, and colistin. MICs to ceftazidime, cefepime, ampicillin/sulbactam, piperacillin/tazobactam, and minocycline were evaluated from 2020 to 2023. Antimicrobial agents were selected according to the European Committee for the Antimicrobial Susceptibility Testing (EUCAST) guidelines, as well as according to the European Centre for Disease Prevention and Control (ECDC) and the Centers for Disease Control and Prevention (CDC) suggestions [15]. Results were evaluated and isolates were defined as susceptible (including susceptible, susceptible increased exposure) and resistant based on EUCAST guidelines [15,16]. MIC to colistin was determined by the broth microdilution method (SensiTest^TM^ Colistin, Liofilchem, Roseto degli Abruzzi, Italy), as recommended [15]. Due to the lack of EUCAST or CLSI clinical breakpoints, MIC to tigecycline was interpreted according to the recommendation of the US Food and Drug Administration, and susceptibility to tigecycline was determined as MIC ≤ 2 μg/mL [17,18,19]. Minocycline, ampicillin-sulbactam, cefepime, ceftazidime, and piperacillin-tazobactam, due to the lack of EUCAST clinical breakpoints, were evaluated according to the CLSI guidelines [20]. Specifically, isolates with MIC to minocycline ≥8 μg/mL [21], ampicillin/sulbactam ≥16/8 μg/mL, cefepime ≥16 μg/mL, ceftazidime ≥16 μg/mL, and piperacillin-tazobactam ≥32/4 μg/mL were classified as non-susceptible (including intermediate and resistant isolates) to respected agents.

### 2.3. MDR/XDR/PDR Definitions

The terms MDR (multidrug-resistant), XDR (extensively drug-resistant), and PDR (pandrug-resistant) were used as described. Specifically, MDR is defined as acquired non-susceptibility to at least one agent in three or more antimicrobial categories among aminoglycosides, antipseudomonal carbapenems, anti-pseudomonal fluoroquinolones, antipseudomonal penicillins plus beta-lactamase inhibitors, extended-spectrum cephalosporins, folate pathway inhibitors, penicillin plus beta-lactamase inhibitors, polymyxins, and tetracyclines. XDR was defined as non-susceptible to at least one agent in all but two or fewer antimicrobial categories (i.e., bacterial isolates remain susceptible to only one or two categories) and PDR was defined as non-susceptible to all agents in all antimicrobial categories [15]. Isolates that were not eligible for categorization as MDR, XDR, and PDR were assigned as susceptible (S).

### 2.4. Statistical Analysis

A statistical analysis was conducted using IBM SPSS Statistics (version 25). Differences between *A. baumannii* and *Acinetobacter* non-*baumannii* spp. (NAB) isolates regarding antimicrobial resistance and department were evaluated using Pearson’s chi-square (chi2) test. Also, alterations related to the number of *A. baumannii* BSI cases per year as well as differentiation between *A. baumannii* isolates resistance patterns (S, MDR, XDR, PDR), tigecycline resistance, and colistin resistance per year and per department were also assessed. Retrieved data were compared using *t*-test or non-parametric Wilcoxon-type test for statistically significant differences. The results were considered statistically significant at *p* < 0.05 and very significant at *p* < 0.001.

## 3. Results

### 3.1. Acinetobacter spp. Isolation

**Figure 1:**
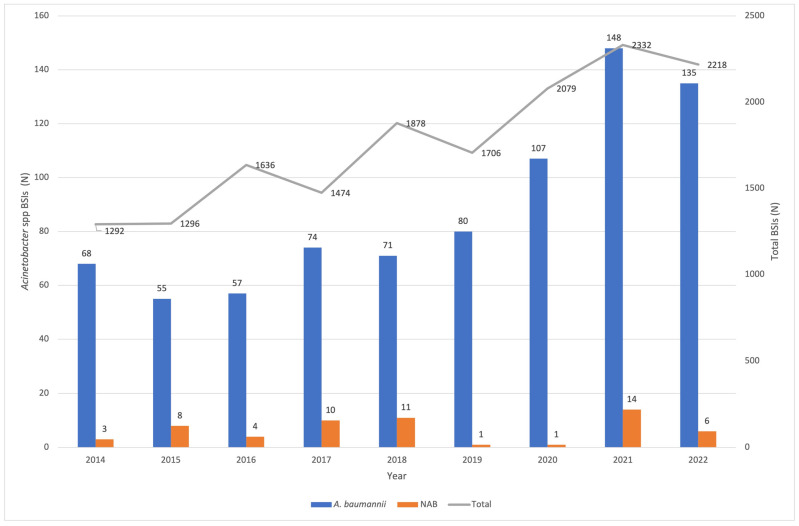
*A. baumannii*, *Acinetobacter* non-*baumannii* (NAB), total BSIs per year, BSIs: bloodstream infections.

During the study period, 15,911 BSIs were recorded. *Acinetobacter* spp. was isolated in 853 cases, accounting for 5.36% of BSIs. The respective cases during the study period are presented on Figure 1. *A. baumannii* was isolated in 795 cases (93.2%), whereas NAB was in 58 cases (6.8%). Specifically, the following species were isolated: *A. lwoffii* (41 cases), *A. haemolyticus* (4 cases), *A. junii* (2 cases), *A. radioresistens* (2 cases), and *A. ursingii* (2 cases), whereas in 7 cases NAB isolates were not identified at species level. In our settings, a significant increase in the number of *A. baumannii* BSIs was recorded in 2020 as compared to previous years (*p* = 0.0012), and a very significant increase in 2021 and 2022 as compared to the period between 2014 and 2019 (*p* ≤ 0.001).

### 3.2. Temporal and Department Distribution of Acinetobacter spp. BSIs

Regarding department distribution, most cases were identified in ICU (394 cases, 46.2%). In MW, 358 cases (42%) were recorded, including 55 cases in hematological patients. In SW, 81 cases (9.5%) were identified, including 32 cases in Neurosurgery. Twenty cases (2.3%) were recorded in NICU and PICUs. Differences were observed regarding department distribution between *A. baumannii* BSI cases and NAB BSI cases. NABs were more commonly isolated in NICU/PICU (4 out of 20 cases), in Haematology–Oncology and Hematopoietic Stem Cell Transplantation Unit (12 out of 55 cases) and in Neurosurgery (5 out of 32 cases) (*p* < 0.001), as compared to other departments. On the other hand, NABs were rarely encountered in ICU (9 out of 395 cases) (*p* < 0.001).

**Figure 2:**
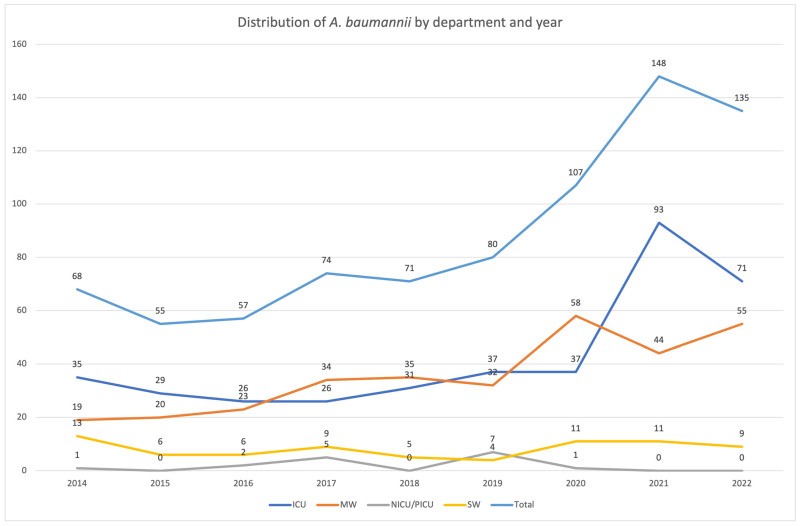
Distribution of *A. baumannii* BSI cases (number) by department and year. ICU: intensive care unit, MW: medical wards, NICU/PICU: neonatal/pediatric Intensive Care Unit, SW: surgical wards.

As shown on Figure 2, an increase in the number of *A. baumannii* BSI cases in ICU was observed in 2021 and 2022, as compared to previous years (*p* < 0.001).

### 3.3. Antimicrobial Resistance Rates of Acinetobacter spp. BSI Isolates

Since NAB isolates exhibited a susceptible phenotype, further discussion on antimicrobial resistance refers only to *A. baumannii* isolates. The resistance rates are presented in Table 1. Specifically, carbapenem-resistance rates exceeded 96% throughout the study period, the only exception being for the year 2016. Resistance to aminoglycosides was also very high, with gentamicin and tobramycin being more active than amikacin. Ciprofloxacin and levofloxacin were inactive in >97.4% of the cases over the period of study, whereas the resistance rate to trimethoprim/sulfamethoxazole was between 63.2 and 94.6%. The resistance to minocycline was lower (74.1%) in 2022 as compared to 2020–2021 (91.9–93%). The opposite trend was observed regarding ampicillin/sulbactam, in 2020 resistance was 73.8%, whereas in 2021 raised to 85.1% and in 2022 to 98.5%.

### 3.4. In Vitro Activity of Tigecycline against A. baumannii BSI Isolates

The resistance to tigecycline demonstrates significant fluctuation. Nevertheless, during the last years of the study (2020–2022), tigecycline resistance increased significantly (*p* < 0.001) and *A. baumannii* isolates with MIC values ≤ 2 μg/mL accounted for 28.5% of all isolates. Moreover, isolates with MIC ≤ 1 accounted for 4.1%, and isolates with MIC ≤ 0.5 accounted *only* for 2.3% of all *A. baumannii* BSI isolates. These data correspond to MIC_50_ = 4 μg/mL and MIC_90_ above 8 μg/mL.

The resistance rates of tigecycline used in the different departments, during the study period (2014–2022) revealed lower resistance rates in the NICU/PICU and Haematology–Oncology Unit, 37.5% and 35.7% respectively; thus, susceptible hosts, such as hematological patients and neonates, are more prone to develop *A. baumannii* tigecycline susceptible bacteremia. High tigecycline resistance rates were observed in ICU (58.2% during 2014–2022); moreover, a significant increase in resistance was observed during the last two years leading to 73.6% resistance rates in 2021 and 83.6% in 2022 (*p* < 0.001). Also, in MW (56% resistance during the study period 2014-22), a significant increase in resistance rate to tigecycline was observed in the last two years, 80% in 2021 and 71.7% in 2022 (*p* < 0.001). An analogous trend was observed in SW (56.3% resistance rate during 2014–2022). There, resistance to tigecycline raised up to 75% in 2021 and 62.5% in 2022.

### 3.5. In Vitro Activity of Colistin against A. baumannii BSI Isolates

**Figure 3:**
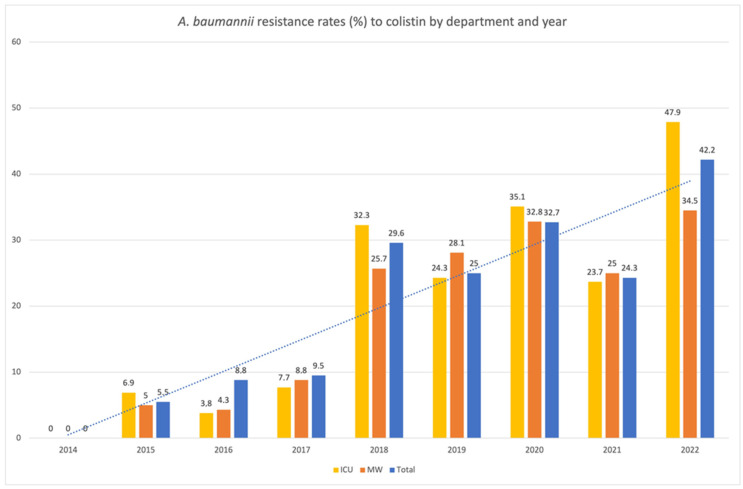
*A. baumannii* resistance rates (%) to colistin by department and year. ICU: Intensive Care Unit, MW: medical wards. Trendline (dashed line) refers to colistin resistance rates (%) of all (total) *A. baumannii* isolates per year.

In our settings, *Acinetobacter* resistance to colistin appeared for the first time in 2015. Afterward, resistance exhibited a growing pattern between 2014 and 2022 (*p* < 0.001), rising to 42.2% in 2022 (Figure 3). This trend was prominent in ICU, MW and SW during the study period (*p* < 0.001). Higher resistance rates were observed in ICU (reaching 47.9% in 2022) and MW (42.2% in 2022). Colistin resistance in SW, including Neurosurgery, was common (44.4% in 2022). Interestingly, low resistance rates were recorded in NICU as well as in hematological wards.

### 3.6. MDR/XDR/PDR Phenotypes of A. baumannii BSI Isolates

Regarding antimicrobial resistance patterns, the vast majority (94.8%) of NABs were tested susceptible in all agents. Only three isolates (5.2%) (one *A. radioresistens* and two *A. lwoffii*) were characterized as MDR/XDR. Specifically, one *A.* lwoffii was MDR and the remaining two isolates were XDR, retaining susceptibility only to colistin and tetracyclines (tigecycline and minocycline). On the other hand, the vast majority (97.7%) of *A. baumannii* isolates are resistant to multiple antimicrobial agents (MDR/XDR/PDR). Thus, *A. baumannii* isolates are more resistant than NAB isolates (*p* < 0.001). During the study period, 4.5% of the isolates was MDR, 84.7% XDR and 8.5% PDR. In addition, a significant increase in PDR isolates was observed during the 2018–2022, (range 10.8–12.2%) as compared to 2014–2017 (0–3.6%) (*p* < 0.001).

## 4. Discussion

Healthcare-associated infections represent a major issue in terms of public-health implications and healthcare costs [22]. The most frequent healthcare-associated infections are those related to invasive medical devices, including central line-associated bloodstream infections and ventilator-associated pneumonia. One of the most involved etiologic agents is *A. baumannii* [22,23]. The major impact of *A. baumannii*-associated infection on the healthcare system can be reliably estimated considering the fact that healthcare-associated infections occur in 4–7% of hospitalizations [22] and that infections caused by *A. baumannii* account for ~2–4% of all healthcare-associated infections globally [4]. Indeed, carbapenem-resistant *A. baumannii* infections are the fourth-leading cause of death, attributable to antimicrobial resistance globally [24].

In our settings, *A. baumannii* accounts for the most *Acinetobacter* spp. BSI cases. Similar findings were retrieved in a national study in Serbia, where *A. baumannii* accounted for 96% of all *Acinetobacter* spp. invasive isolates [11]. On the contrary, in a surveillance study conducted in England in 2020, 30% of *Acinetobacter* spp. BSI isolates were identified as *A. lwoffii* and only 19.5% as *A. baumannii* [25]. In another study in Germany, only 44% of all *Acinetobacter* spp. were assigned as *A. baumannii* complex [26].

Almost 90% of cases (88.2% of *Acinetobacter* spp. BSIs and 88.7% of *A. baumannii*) were documented in ICU and MWs. Hospitalization in ICU, along with other known risk factors for *A. baumannii* infection, is a common predisposing factor for invasive infections in these patients. Common features in ICU patients involve prolonged hospital stay, immunosuppression, invasive procedures (central venous catheterization, mechanical ventilation, surgery), enteral feeding, severity of illness, and the use of third-generation cephalosporins/carbapenems [2,23]. Apart from ICU, *Acinetobacter* spp. BSI cases exhibited a high incidence in MWs. We should denote that a common practice for ICU patients before hospital discharge is to be transferred to MWs for rehabilitation purposes. Moreover, during the COVID-19 epidemic, MWs housed severely ill patients, similar to those hospitalized in intensive care units.

Recent data from the European Antimicrobial Resistance Surveillance Network (EARS-Net) revealed a large increase (+57%) in *Acinetobacter* spp. BSIs in the European Union and European Economic Area in the first years of the COVID-19 pandemic (2020–2021), as compared to 2018–2019 [3]. Most isolates were resistant to carbapenems, originated from patients in ICUs, hospitalized in countries with confirmed ≥50% carbapenem-resistance in *Acinetobacter* spp. [3]. In accordance with these data, in our settings an increase in the net number of *A. baumannii* cases from 151 cases in 2018–2019 to 255 cases in 2020–2021 was documented, corresponding to an increase of 69%. The total number of BSIs in the same periods increased from 3584 to 4411, corresponding to an increase of 23%. The observed increasing trends were probably driven by the profound impact of the COVID-19 pandemic on hospital care, which increased the number of patients at risk of *Acinetobacter* spp. BSI, and in problematic implementation of infection-control measures in overcrowded ICUs with a low personnel/patient ratio [3]. Data supporting this postulation is a similar increase in “low pathogenicity” pathogens, such as *Candida* spp. [27]. Prolonged hospitalization in the ICUs has been associated with an increased possibility of developing bacterial co-infection, especially with the multidrug resistant (MDR) *A. baumannii* in critically ill COVID-19 patients [7]. Also, respiratory viral infections, such as SARS-CoV-2, predispose patients to bacterial co-infections and secondary infections [7].

Differences in resistance rates between NAB and *A. baumannii* are prominent. In the group of NAB isolates, three out of 58, exhibited an MDR/XDR phenotype. The low resistance rates concerning NAB, mainly *A. lwoffii*, as the predominant non-*baumannii* species, is in accordance with another study that also describes low resistance percentages, between 0 and 8% in 2020, for *A. lwoffii* [25]. Nevertheless, one *A. lwoffii* was characterized as MDR and one *A*. *radioresistens* and one *A*. *lwoffii* were XDR, retaining susceptibility only to colistin and tetracyclines (tigecycline and minocycline). The occurrence of resistant phenotypes among NAB isolates has also been reported in other publications [28,29]. The scenario that these isolates acquired resistance genes from *A. baumannii* isolates that thrive in a hospital environment seems probable. Moreover, the fact that hospital-acquired multidrug-, extensively drug-, or even pandrug-resistant *A. baumannii* isolates colonize discharged patients and may be dispersed in the environment is worrisome [30]. On the other hand, the overuse of antibiotics in poultry, livestock, and agriculture may result in the emergence of resistance genes in “less pathogenic” NAB isolates [31]. Specifically, *A. radioresistens* is considered the progenitor of the *bla*_OXA-23_-like genes that are identified in carbapenem-resistant *A. baumannii* isolates [32]. Thus, the interplay between *Acinetobacter baumannii* and related, mostly environmental, species, that can serve as reservoirs of resistance determinants may contribute to the prevalence of MDR pathogens.

High resistance rates to carbapenems are evident in our study. Conversely, low resistance rates have been documented in other countries, such as England and Germany, where isolates resistant to carbapenems accounted for 9–13% [25] and 8.1% [26], respectively. In the latest World Health Organization (WHO) and European Centre for Disease Prevention and Control (ECDC) report, the percentages of carbapenem-resistant *Acinetobacter* spp. varied widely within Europe in 2020; specifically, among 38 countries and areas reporting data, three countries (Ireland, The Netherlands, and Norway) reported occurrence rates of less than 1%, whereas 21 countries reported rates of 50% or higher, mostly in southern and eastern Europe [33]. Notably, the prevalence of carbapenem-resistant *A. baumannii* at rates of over 80% has been reported in all Balkan countries [34]. Specifically, in Croatia, carbapenem-resistance is almost 97% [34], in Bulgaria 100% [35], and in Serbia 93.7% [11].

Regarding aminoglycosides, in our settings tobramycin and gentamicin were more active in vitro, as far as *A*. *baumannii* isolates exhibited resistance rates of 86.5% and 87.9% to the above agents, respectively. On the contrary, the resistance rate to amikacin was as high as 92.5%. In a study conducted in Bulgaria, similar results were obtained; resistance rates regarding tobramycin and gentamicin were 86.3% and 89%, respectively, whereas resistance to amikacin was higher, 98.6% [35]. Recent data showed that amikacin non-susceptibility correlated with overproduction of *adeB*, whereas an analogous trend was not documented concerning gentamicin and tobramycin [36].

Tigecycline possesses a broad spectrum of activity against aerobic and anaerobic bacteria and is indicated for the treatment of complicated skin and skin structure infections, complicated intra-abdominal infections, and community-acquired bacterial pneumonia [37]. In our study, more than half of *A. baumannii* strains isolated between 2015 and 2020 exhibited an MIC below or equal to 2 μg/mL. Thereafter, isolates with MIC ≤ 2 μg/mL represented only 28.46% of all isolates, corresponding to MIC_50_/MIC_90_ 4/8. In accordance with our results, in a recent multicenter study from Greece (not including University Hospital of Patras), tigecycline MIC_50_/MIC_90_ during 2020–2021 was estimated to be 4/8 μg/mL [38]. High dose of tigecycline is recommended in order to maintain satisfactory serum concentrations in *Acinetobacter* bacteremias caused by isolates possessing MICs ≤ 0.5 μg/mL or at least ≤1 μg/mL, when alternative treatments are not possible [18,39]. In our settings, isolates owning MIC of tigecycline ≤1 μg/mL accounted for 4.1% of all *A. baumannii* retrieved between 2020 and 2022. Thus, tigecycline could constitute an effective treatment option only for a minority of BSI cases.

Colistin is a cationic peptide that binds to and destabilizes the lipopolysaccharide on the outer membrane of Gram-negative bacilli. Colistin (polymyxin E)-based therapy, which is often used in combination, has been described as a last resort for the treatment of MDR *A. baumannii*. Nevertheless, colistin-resistant *A. baumannii* strains have been reported in various regions. In a recent study performed at a national level in England, 20% of tested isolates were colistin resistant [25]. In Croatia, colistin resistance was reported in 2% of carbapenem-resistant *A. baumannii* [34], whereas in Serbia, 3.94% of isolates were confirmed as colistin-resistant [11]. In Italy, colistin-resistant isolates accounted for 18.7% of all isolates, showing a 10% increase during a four-year period [40]. In Egypt, colistin-resistant isolates have been reported at rates of 52.9% [41]. In another recent multicenter study from Greece, during 2020–2021, colistin-susceptible isolates were only 15.5% [38]. In our setting, colistin resistance demonstrates an increasing pattern.

Data from our institution reveal that the overall administration of tigecycline increased during the study period, while the consumption of colistin remained stable [42]. Despite stable colistin consumption, colistin resistance increased during the study period. In contrast, colistin resistance remained stable during 2015 to 2021, in respect to *Klebsiella pneumoniae*, another common and multidrug-resistant pathogen that thrives in ICU [42]. Analogous ambiguous observation was made in a retrospective study in Italy that presented the results of an antimicrobial stewardship program [40]. There, most resistance indexes showed reduction apart from colistin resistance of *A. baumannii* that increased per 10% [40]. Nevertheless, colistin represent a “standard” used regimen for treatment of *A. baumannii* BSIs, even associated to pandrug-resistant isolates [43]. Increased use of polymyxins to order to treat XDR/PDR *A. baumannii* may be responsible for the prevalence of colistin-resistant isolates.

Increased tigecycline consumption correlates with increasing resistance rates. We should note that in the first year of the study, 2014, resistance to tigecycline was 55.9%. Afterward, an increasing trend was recorded, leading to 77% resistance rates in 2022.

MDR/XDR/PDR *A. baumannii* isolates represented the vast majority (97.7%) of isolates. High rates of MDR *Acinetobacter* spp. were reported in Serbia (95.9%) and Croatia (95.1%), whereas the respective value for Kosovo was 71.2% [35]. The very high genetic plasticity of *A. baumannii* allows the easy acquisition of resistance genes via mobile elements [7]. In addition to intrinsic mechanisms, enzymatic hydrolysis, reduced membrane permeability, overexpression of efflux pumps, and modifications in antibiotic binding targets contribute to antibiotic resistance [7].

The large increase in MDR, XDR, or even PDR *Acinetobacter baumannii*-associated nosocomial BSIs calls for reinforced application of preventive and response actions. Treatment of these infections remains a great challenge for clinicians. Effective treatment may require a personalized approach that incorporates host factors as well as local molecular epidemiology records [24]. The preferred regimen consists of high-dose ampicillin-sulbactam, in combination with either polymyxin B, or tigecycline dependent on PK-PD optimized dosing, susceptibility testing results, and the site of infection; moreover, a third agent can be added in cases with delayed clinical responses or recurrent infections [24]. Newly developed β-lactam agents, like cefiderocol and durlobactam, in combination with sulbactam, the use of phage technology [44], and monoclonal antibodies [24], represent novel approaches in the era of antibiotic shortage.

The strengths of the present study are: (a) The representative population of patients included in the study (University hospital of Patras is a referral tertiary hospital of Southwestern Greece); (b) the long period of study, and; (c) the large number of isolates. All these data enable us to draw safe conclusions. The limitations of the present study include the absence of clinical data to identify potential risk factors for the development and predictors of mortality of these infections. Another limitation was the absence of molecular studies of *Acinetobacter* spp. isolates, to assess genetic relatedness of isolates.

Awareness/knowledge of the current epidemiological status is the most important step in the race to combat the spread of multidrug-resistant *A. baumannii*. The implementation of a national surveillance action plan and the establishment of a prompt detection system for the detection of the onset of epidemic clusters and significant events within healthcare facilities is substantial [22]. As far as incidence of nosocomial infections is a quality-of-care index, measures should be applied to minimize infectious diseases spread within hospitals, including, but not limited to hand washing, sterilization, disinfection, isolation protocols, a wiser use of antibiotics [22]. The health facility must take care not only to introduce preventive practices and educate healthcare staff, but also, and most importantly, to verify the implementation of these practices [22].

## 5. Conclusions

*A. baumannii* causes severe nosocomial infections including bloodstream infections, mainly in critically ill patients. The establishment of *A. baumannii* that exhibits multidrug- or extensively drug-resistance patterns is a matter of great concern for the healthcare community at local as well as at international level. The emergence of pandrug-resistant *A. baumannii* isolates poses a global threat to human health. It signifies the urgent need for efficient antimicrobial stewardship, for control spread measures, and early recognition and dealing with epidemic events.

## Figures and Tables

**Table 1:** Antimicrobial resistance (%) of *A. baumannii* during 2014–2022.

	AMK	GEN	TOB	CIP	LEV	IPM	MEM	TGC	SXT	CAZ	CEF	SAM	TZP	MIN
**2014**	97.1	91.2	89.7	97.1	97.1	98.5	98.5	55.9	82.4					
**2015**	98.2	92.7	96.4	96.4	96.4	96.4	98.2	27.3	76.4					
**2016**	84.2	78.9	78.9	89.5	89.5	91.2	93.0	43.9	63.2					
**2017**	97.3	94.6	90.5	97.3	97.3	97.3	97.3	32.4	94.6					
**2018**	95.8	97.2	95.8	98.6	98.6	98.6	98.6	45.1	91.5					
**2019**	100	93.2	91.5	100	100	100.0	100.0	35.6	93.2					
**2020**	92.5	86.9	76.6	98.1	98.1	99.1	98.1	59.8 *	85.0	100.0	100.0	73.8	96.3	93.0 ^+^
**2021**	93.9	88.5	88.5	98.0	98.0	96.6	96.6	75.0 *	70.9	99.3	100.0	85.1	96.6	91.9
**2022**	96.3	91.1	94.1	98.5	98.5	98.5	99.3	77.0 *	93.3	99.3	98.5	98.5	98.0	74.1
**2014–2022**	**92.5**	**87.9**	**86.5**	**97.4**	**97.4**	**97.6**	**95.2**	**56.1**	**83.5**	**95.9**	**95.4**	**86.7**	**92.5**	**85.6**

AMK: amikacin, GEN: gentamicin, TOB: tobramycin, CIP: ciprofloxacin, LEV: levofloxacin, IPM: imipenem, MEM: meropenem, TGC: tigecycline, SXT: trimethoprim/sulfamethoxazole, CAZ: ceftazidime, CEF: cefepime, SAM: ampicillin/sulbactam, TZP: piperacillin/tazobactam, MIN: minocycline, * *p* < 0.001, ^+^: data available for 86 out of 107 isolates.

## Data Availability

The data included in this manuscript cannot be shared publicly, due to the need to protect the privacy of the included subjects. Data may be shared upon reasonable request to the corresponding author.
